# Cereal aphids differently affect benzoxazinoid levels in durum wheat

**DOI:** 10.1371/journal.pone.0208103

**Published:** 2018-12-03

**Authors:** Reut Shavit, Zhaniya S. Batyrshina, Nitsan Dotan, Vered Tzin

**Affiliations:** 1 French Associates Institute for Agriculture and Biotechnology of Drylands, Jacob Blaustein Institutes for Desert Research, Ben-Gurion University of the Negev, Sede Boqer Campus, Israel; 2 Ilse Katz Institute for Nanoscale Science and Technology, Ben-Gurion University of the Negev, Beer-Sheva, Israel; Huazhong University of Science and Technology, CHINA

## Abstract

Aphids are major pests in cereal crops that cause direct and indirect damage leading to yield reduction. Despite the fact that wheat provides 20% of the world’s caloric and protein diet, its metabolic responses to aphid attack, in general, and specifically its production of benzoxazinoid defense compounds are poorly understood. The objective of this study was to compare the metabolic diversity of durum wheat seedlings (*Triticum turgidum* ssp. durum) under attack by three different cereal aphids: i) the English grain aphid (*Sitobion avenae* Fabricius), ii) the bird cherry-oat aphid (*Rhopalosiphum padi* L.), and iii) the greenbug aphid (*Schizaphis graminum* Rondani), which are some of the most destructive aphid species to wheat. Insect progeny bioassays and metabolic analyses using chromatography/Q-Exactive/mass spectrometry non-targeted metabolomics and a targeted benzoxazinoid profile were performed on infested leaves. The insect bioassays revealed that the plants were susceptible to *S*. *graminum*, resistant to *S*. *avenae*, and mildly resistant to *R*. *padi*. The metabolic analyses of benzoxazinoids suggested that the predominant metabolites DIMBOA (2,4-dihydroxy-7-methoxy-1,4-benzoxazin- 3-one) and its glycosylated form DIMBOA-glucoside (Glc) were significantly induced upon both *S*. *avenae*, and *R*. *padi* aphid feeding. However, the levels of the benzoxazinoid metabolite HDMBOA-Glc (2-hydroxy-4,7-dimethoxy-1,4-benzoxazin-3-one glucoside) were enhanced due to the feeding of *S*. *avenae* and *S*. *graminum* aphids, to which Svevo was the most resistant and the most susceptible, respectively. The results showed a partial correlation between the induction of benzoxazinoids and aphid reproduction. Overall, our observations revealed diverse metabolic responses of wheat seedlings to cereal aphid feeding.

## Introduction

Wheat was first domesticated more than 10,000 years ago, making it one of the earliest domesticated crops, and it still remains the most widely grown crop in the world [1,2]. Its global production throughout the world produces 749 million tons of grain that provides 20% of the world population’s calories and protein [3]. With continuing population growth, the demand for food is predicted to increase by 40% by the year 2050, triggering a need to increase crop yield. Pests are one of the main causes of crop loss, with an average of 15% crop loss worldwide [4]. In tropical areas, the loss can be massive, up to 50% [5]. The most economically significant pests are found in the aphid family (Hemiptera: Aphididae), which comprises approximately 5,000 species distributed globally [6]. Aphids cause enormous yield losses due to the direct and indirect crop damage they inflict [7]. Aphid feeding causes a reduction in nutrients and photosynthetic efficiency while transmitting toxins in their saliva [8–12]. Additionally, aphids are responsible for transmitting about 40% of all plant viruses, including the most harmful of wheat viruses, the barley yellow dwarf virus (BYDV) [13,14].

Several of the most economically important aphids associated with wheat are extensively described by Blackman and Eastop (2000), including the English grain aphid (*Sitobion avenae* Fabricius), the bird cherry-oat aphid (*Rhopalosiphum padi* L.), the greenbug aphid (*Schizaphis graminum* Rondani), the rose-grain aphid (*Metopolophium dirhodum*), and the Russian wheat aphid (*Diuraphis noxia* Kudjumov) [15]. These aphids attack cereal crops and are commonly called “cereal aphids” [16], though they may differ in their morphology and physiology. For example, in Brazil, it was reported that *R*. *padi* is the species most frequently found in wheat fields, especially during winter, followed by *S*. *avenae*, which is commonly observed during the wheat heading stage [17]. Additionally, *R*. *padi* is relatively stable in abundance and is also the most efficient in transmitting viruses in southern Brazil [18], as well as the most polyphagous with a host range of well over 100 species [19]. The aphids *S*. *graminum* have been shown to cause unique damage to wheat by generating yellow or red leaf spots, which may be caused by the secretion of enzymes in the saliva that break down cell walls and chloroplasts in susceptible plants [20].

The main approach to controlling the aphid population in crops is through insecticidal treatments that while capable of inducing plant resistance to aphids after prolonged use, can also have hazardous ecological effects [7,21]. When taking climate change and the introduction of wheat to new regions into account, the negative impact of aphids on wheat yield could potentially increase [4,22,23]. In response to herbivore attacks, plants produce specialized metabolites to reduce damage and preserve their fitness [24,25]. Plant responses include large defense mechanisms, such as the synthesis of deterrent molecules (i.e., benzoxazinoids), the emission of volatiles (i.e. terpenoids), callose formation and deposition, and the biosynthesis of flavonoids and cell wall compounds [11,25]. During the 1960s, researchers identified the function of hydroxamic acids (HAs) or benzoxazinones in resistance to insect herbivores, bacteria, fungi, nematodes, mites and insects [26–32]. Previous studies suggested that the benzoxazinoids’ protective effects are due to anti-feeding properties driven by inhibition of the insect digestive proteases responsible for detoxification and salivation [28,33,34], and by regulation of callose formation [32,35]. This class of metabolites is present in cereals such as maize, wheat, rye, and several wild barley species [36–39], while their synthesis either occurs constitutively in young seedlings, which varies between the plant species [40,41], or is induced by insect feeding [42]. The main toxic benzoxazinoid abundant in maize and wheat is DIMBOA (2,4-dihydroxy-7-methoxy-1,4-benzoxazin- 3-one), which is stored in the cell vacuoles in a nontoxic form as DIMBOA-glucoside (Glc) [17,21,41], and can be hydrolyzed by a β-glucosidase enzyme when the tissue is damaged. Although the benzoxazinoid compounds, such as DIMBOA and its glycosylated form DIMBOA-Glc, as well as 2-hydroxy-4,7-dimethoxy-1,4-benzoxazin-3-one glucoside (HDMBOA-Glc) and 6-methoxy-benzoxazolin-2-one (MBOA), were reported in tetraploid and hexaploid wheat genotypes [17,41,43,44], thus far, wheat biosynthetic genes orthologous to maize *Bx7*and *Bx10-Bx14* have not yet been found in this plant [45,46]. Additionally, *Bx6* is only putatively annotated in the diploid goat grass wheat (DD, *Aegilops tauschii*; NCBI accession number XM_020325695) and bread wheat (AABBDD, *Triticum aestivum*; NCBI accession number JW033819) [47].

In this study, we investigated the constitutive and inducible benzoxazinoid levels in wheat seedlings in response to cereal aphid infestation and the differences in aphid reproduction [41]. We explored the metabolic responses of the durum wheat seedling (*Triticum turgidum* ssp. durum) accession named Svevo to three selected aphid species: i) the grain aphid (*Sitobion avenae* Fabricius), ii) the bird cherry-oat aphid (*Rhopalosiphum padi* L.), and iii) the greenbug aphid (*Schizaphis graminum* Rondani). These aphids constitute major wheat pests and are widely distributed in wheat-producing areas, with some effects driven by the geographic region [48,49]. The interaction between aphid and wheat triggers plant defense responses, which affect the plant metabolism including the biosynthesis of specialized metabolites which then affect aphid reproduction. Pereira *et al*. (2017) recommended assessing wheat plant resistance to aphids using three characteristics: i) antixenosis (negative effect on the insect acceptance), ii) antibiosis (negative effect on the insect physiology *i*.*e*. preproduction), and iii) tolerance (ability to cope with the attack by the insects whilst sustaining only a small reduction in some characteristic, *i*.*e*. yield) [17]. Therefore, we applied the same number of aphids (10 adults) and counted the progeny (antibiosis) on the same wheat genotype. We investigated the diversity and abundance of benzoxazinoids in the young leaf tissue (seedlings) of durum wheat, and we evaluated whether DIMBOA, DIMBOA-Glc, and HDMBOA-Glc compounds are correlated with antibiosis effects on aphid reproduction. This research expands the knowledge of defense mechanisms in durum wheat against aphid attacks, knowledge that can lead to improved natural wheat resistance, resulting in a decrease of yield losses due to aphid damage.

## Materials and methods

### Plant growth conditions and aphid bioassays

Durum wheat plants were grown in a Conviron walk-in growth chamber at 23 °C with a 16:8 hr light: dark cycle and 180 µmol m^-2^ s^-1^ light intensity. Three aphid species were tested: *Schizaphis graminum* (greenbug), *Sitobion avenae* (English grain aphid), and *Rhopalosiphum padi* (bird cherry-oat aphid). Aphid colonies were maintained on cultivated barley (*Hordeum vulgare*) in a walk-in growth chamber at 20 °C with a 18:6 hr light: dark cycle [50]. Ten-day-old plants were used for aphid bioassays (second leaf emergence to approximately 15 cm). For whole-plant, non-choice aphid bioassays, ten adult aphids were confined on ten-day-old plants using micro-perforated polypropylene bags (15 cm × 61 cm; http://www.pjpmarketplace.com) [21,44]. After 96 hr of infestation, the nymphs and adults were counted to evaluate reproduction.

### Untargeted and benzoxazinoid metabolite analyses using liquid chromatography/mass spectrometry

To measure wheat metabolites, approximately 10 cm of aphid-fed second leaf tissue was collected in parallel with control leaves without aphids from the same non-choice aphid bioassay described above. For non-targeted metabolite assays, frozen powder ground from fresh tissue was weighed in a 1.5-ml tube, and extraction solvent including methanol/double-distilled water/formic acid, 80:19.9:0.1, v/v/v was added in a 1:3 ratio to each leaf sample. After a brief vortex, the tubes were shaken for 40 min at 4 °C, and centrifuged for 5 min at 14 000 g. The samples were filtered through a 0.22-μm filter plate (EMD Millipore Corp., Billerica, MA, USA) by using a centrifuge at 2000 g for 3 min. The supernatant was diluted 1:10 with the extraction solvent, and the solvent was subsequently transferred to an HPLC glass vial [10]. For a liquid chromatography/time-of-flight/mass spectrometry (LC/TOF/MS) non-targeted metabolite assay, the separation was performed using a Dionex UltiMate 3000 Rapid Separation LC System attached to a diode array detector and a Thermo Q-Exactive mass spectrometer (LC/QE/MS; Thermo Scientific). The samples were separated on an Acclaim C18 reverse-phase column (Thermo Scientific) at the flow rate of 0.5 ml min^-1^, using a gradient flow of 0.1% formic acid in LC-MS-grade water (eluent A) and 0.1% formic acid in acetonitrile (eluent B) with conditions as previously described [51]. Raw mass spectrometry data files were processed using the XCMS [52], and CAMERA [53] software packages for R, and the negative ionization dataset was transferred to Microsoft Excel. For targeted benzoxazinoid identification, chromatographic peaks were compared with the retention time, accurate mass and UV spectrum of standards of DIMBOA, DIMBOA-Glc, and HDMBOA-Glc [39], which were provided by Gaetan Glauser (University of Neuchatel, Neuchatel, Switzerland; S5 Table).

For measuring the benzoxazinoid levels, leaf tissue from 15-day-old Svevo plants was harvested, and the benzoxazinoids were extracted following the same method described above. The extract was analyzed on a Dionex UltiMate 3000 Rapid Separation LC System using a C18 reverse-phase Hypersil GOLD column (Thermo Fisher Scientific) using the same conditions described above. We used the authentic standard of DIMBOA (Toronto Research Chemicals, Toronto, Canada), as well as DIMBOA-Glc, DIM2BOA-Glc, HDMBOA-Glc and HDM2BOA-Glc, from a plant crude extract that was confirmed with a UV spectrum for metabolites identification and quantification (the crude extract was provided by Matthias Erb, University of Bern, Switzerland). By using calibration curves, we were able to confirm the contents of DIMBOA, DIM2BOA-Glc, and HDMBOA-Glc in the leaf tissues.

### Statistical analyses

Data for the principal component analysis (PCA) plot was normalized as follows: an average of each parameter (mass signals) was calculated across all samples (treated and untreated), and each individual parameter was divided by its average and subjected to a log 2 value [54]. The normalized values were plotted with the MetaboAnalyst 3.0 software using the following parameters: missing value estimation: remove features with more than 50% missing values and replace by a small value (half of the minimum positive value in the original data), data filtering of interquartile range, and no further data normalization transformation or scaling [55]. Venn diagrams were made using the Venny 2.1.0 drawing tool http://bioinfogp.cnb.csic.es/). Statistical comparisons for Student’s *t*-tests with a false discovery rate (FDR) were calculated by TMEV, a Multiple Experiment Viewer tool (http://mev.tm4.org/), and an analysis of variance (ANOVA) was performed using JMP Pro 12 (SAS; www.jmp.com).

## Results

### Evaluating durum wheat resistance to cereal aphids by measuring reproduction

**Fig 1:**
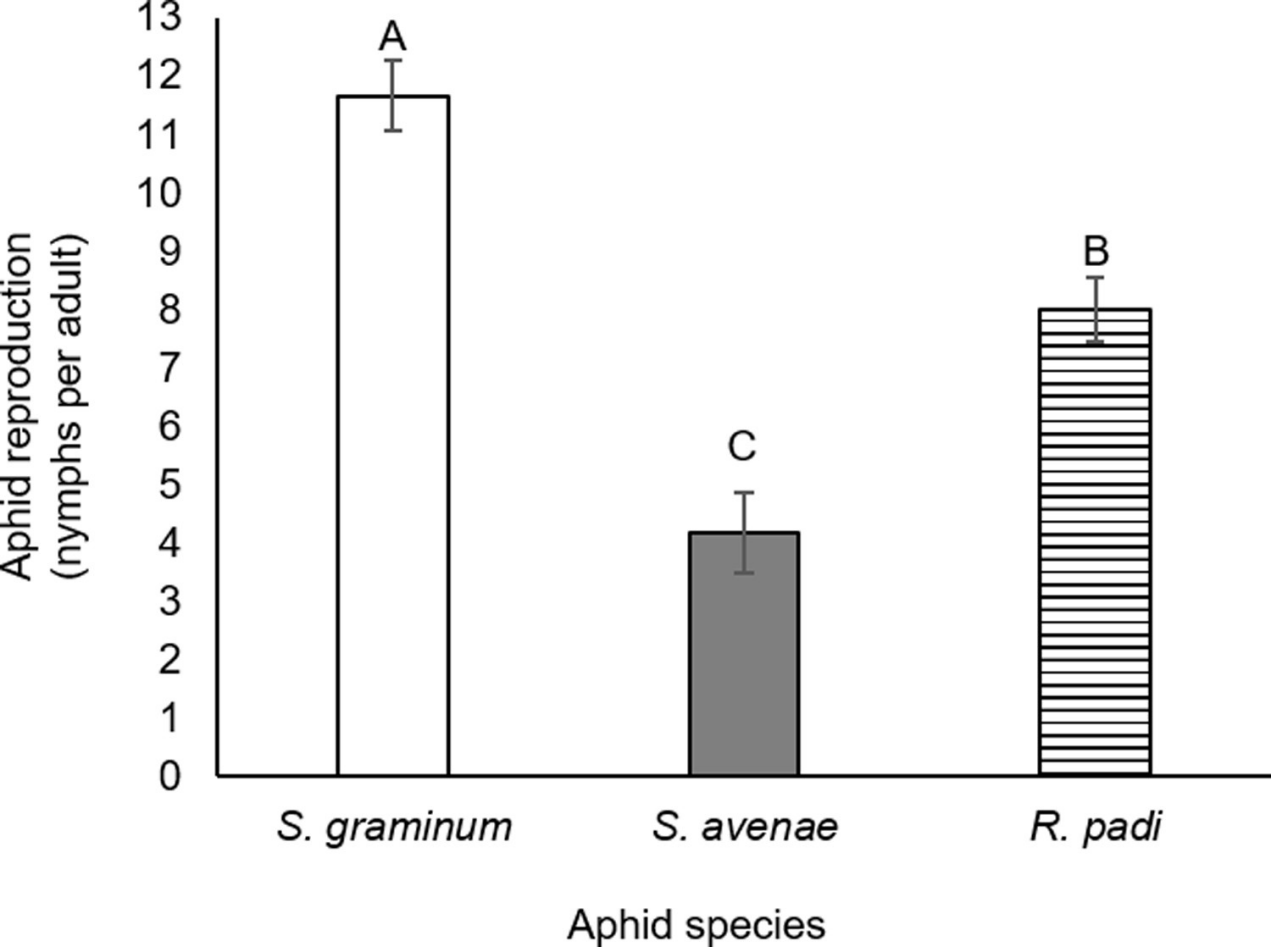
Aphid production using whole-plant, non-choice aphid bioassays infesting durum wheat for 96 h (mean +/- SE). Different letters above bars indicate significant differences, *P* value < 0.05, ANOVA followed by paired Student’s *t*-test (n = 8).

The cereal aphids *Rhopalosiphum padi*, *Schizaphis graminum*, and *Sitobion avenae* are three of the most damaging aphid species to wheat [15,56]. To evaluate the effect of these aphid species on durum wheat, we used a whole cage bioassay applying ten adult aphids. After 96 hr of infestation, the number of nymphs per adults was counted, and leaf tissue was collected for further metabolic analysis (as described below). As shown in Fig 1, the major findings regarding the resistance of Svevo wheat to the aphids are the following: the plants were susceptible to *S*. *graminum*, resistant to *S*. *avenae*, and mildly resistant to *R*. *padi*. These results suggest that each of the three cereal aphid species may trigger the plant defense responses in slightly different manners or to different extents.

### Measuring the impact of cereal aphid feeding on wheat metabolism

**Fig 2:**
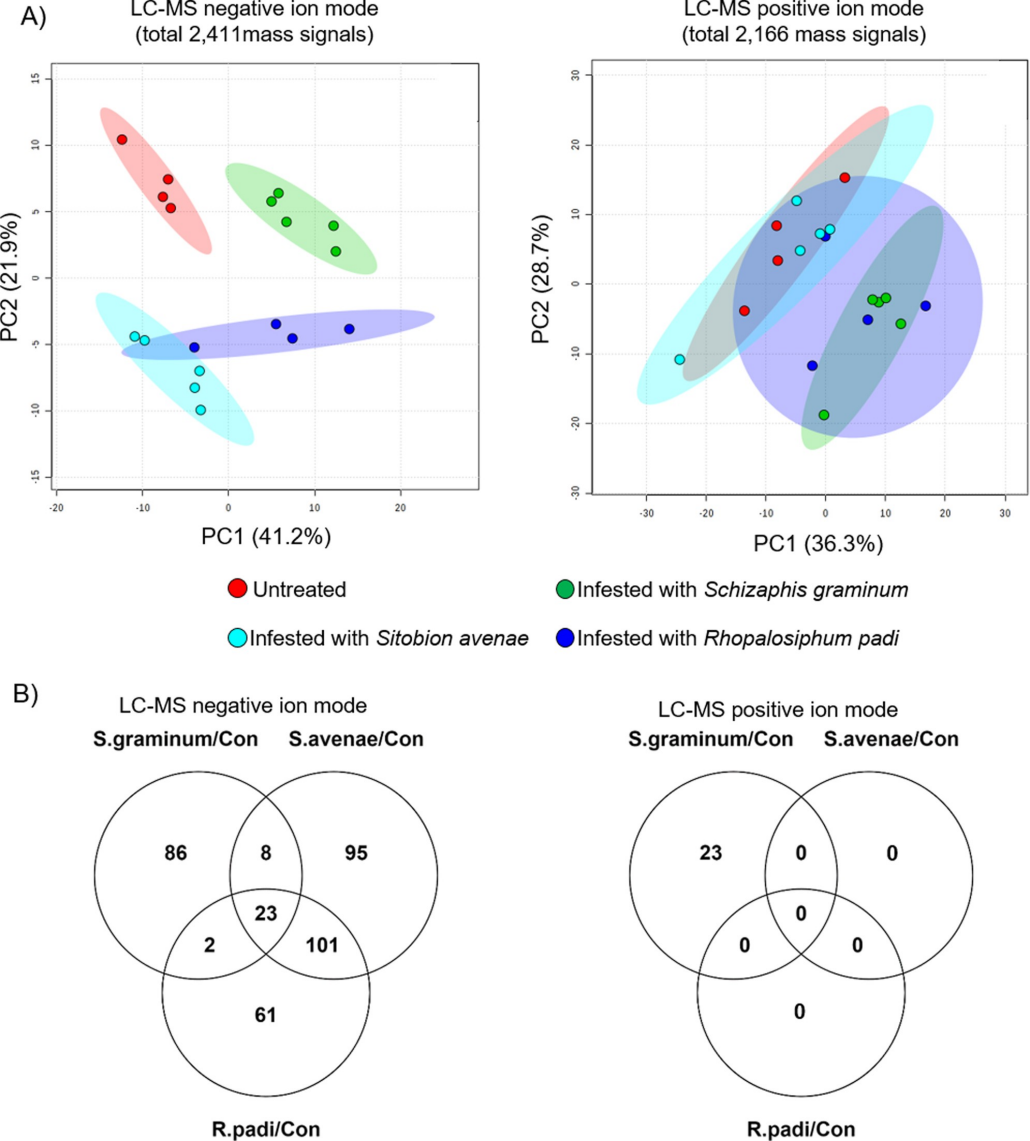
An untargeted metabolic overview of durum wheat infested with *S*. *graminum*, *S*. *avenae* and *R*. *padi* for 96 hr. A) PCA plots of features from negative (2,411 ESI) and positive (total 2,166 ESI) ion modes illustrated by using Metaboanalyst software (n = 4–5). B) Venn diagram illustrating the number of total features significantly up- or down-regulated by cereal aphid treatment; negative ion mode (left) and positive ion mode (right). *P* value < 0.05 FDR and fold change > 2 or < 0.5.

To explore the metabolic diversity of wheat in response to attacks by different cereal aphids, we performed whole-plant, non-choice aphid bioassays (as described in above). After 96 hr of infestation, we removed the aphids and harvested the leaf tissue from the second leaf tip (approximately 10 cm). First, we performed an untargeted metabolic analysis using a liquid chromatography/time-of-flight/mass spectrometry (LC/QE/MS) platform (S1–S5 Tables). The raw mass spectrometry data files were processed using the XCMS, and feature (mass peaks) retention times and m/z were calculated [52]. The feature levels of the negative ion mode were used to conduct a principal component analysis (PCA; Fig 2A). Samples from each treatment, as well as the control (untreated), clustered with one another, which indicates that all the biological replicates of each genotype were clustered together, highlighting the reproducibility of the experiment. The PCA plot of the negative ion mode (components 1 and 2 explained 63.1% of the variance) showed that samples from the *S*. *avenae* and *R*. *padi* treatments clustered furthest from the control samples, indicating that a large change in the metabolome occurred after the onset of these aphids. Also, these results revealed that the Svevo leaves treated with *S*. *graminum* aphids were clustered separately from the other treatments. The PCA plot of the positive ion mode (components 1 and 2 explained 65.0% of the variance) indicated that only the samples of *S*. *graminum-*infested wheat were clustered separately from the untreated plants. Overall, this suggested that in response to aphid feeding, the leaf metabolic profiles were divided into two groups: i) *S*. *avenae* and *R*. *padi*, and ii) *S*. *graminum*.

The distribution of the total significant up and down-regulated features was calculated for each treatment and is presented in Venn diagrams (Fig 2B and S2 and S4 Tables). For the negative ion mode, a total of 376 features were up- or down-regulated in at least one of the treatments, 119 by *S*. *graminum*, 227 by *S*. *avenae*, and 187 by *R*. *padi* (*P* value < 0.05, FDR). Although a unique set of features were altered in each treatment, a small number of features were modified by all treatments (23 features). Relatively large numbers of features were significantly affected upon *S*. *avenae* and *R*. *padi* feeding with a large number of overlapping mass signals (101 features). For the positive ion mode, the only significantly altered features were detected in the leaf tissue after the *S*. *graminum* infestation, suggesting that most of the differentially altered metabolites were detected using the negative ion mode.

**Fig 3:**
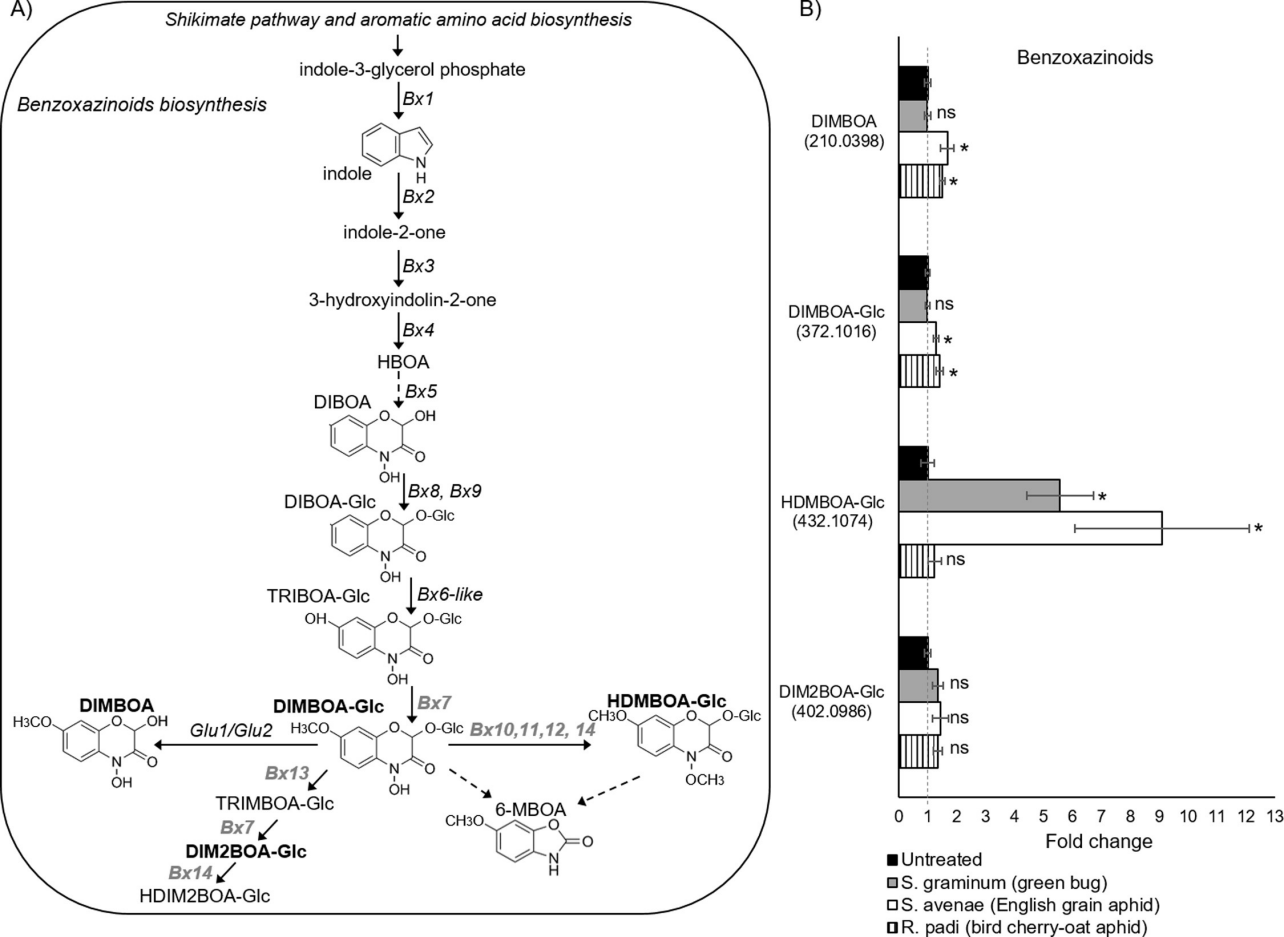
Effects of aphid feeding on benzoxazinoid levels after 96 hr of infestation. A) Schematic representation of benzoxazinoid biosynthesis showing the main enzymatic reactions and intermediates. In grey are the names of genes and metabolites that are detected in maize but are still unknown in wheat. In bold are the four benzoxazinoids that are presented in this research. B) DIMBOA, DIMBOA-Glc, HDMBOA-Glc and DIM2BOA-Glc levels modified under the attack of three cereal aphid species relative to the untreated control. An asterisk indicates the values that were determined by the Student’s t-test to be significantly different (*P* value < 0.05) from the untreated control (n = 4–5). ns: not significant.

Both the PCA clustering pattern and the Venn diagrams reveal the massive metabolic differences caused by the aphid species, with the response to *S*. *graminum* being the most different. Previous studies indicated that in response to *R*. *padi* and *S*. *avenae* infestation, there is mainly a reduction in plant growth and grain yields, whereas *S*. *graminum* feeding also causes plant chlorosis and necrotic spots at the feeding site [20,57]. Therefore, the observed differences in Figs 1 and 3B may be due to the feeding damage generated by *S*. *graminum* infestation. Overall, these results lead us to the conclusion that the metabolic responses of the plant rely on metabolites that confer different modes of resistance to aphid attacks.

**Fig 4:**
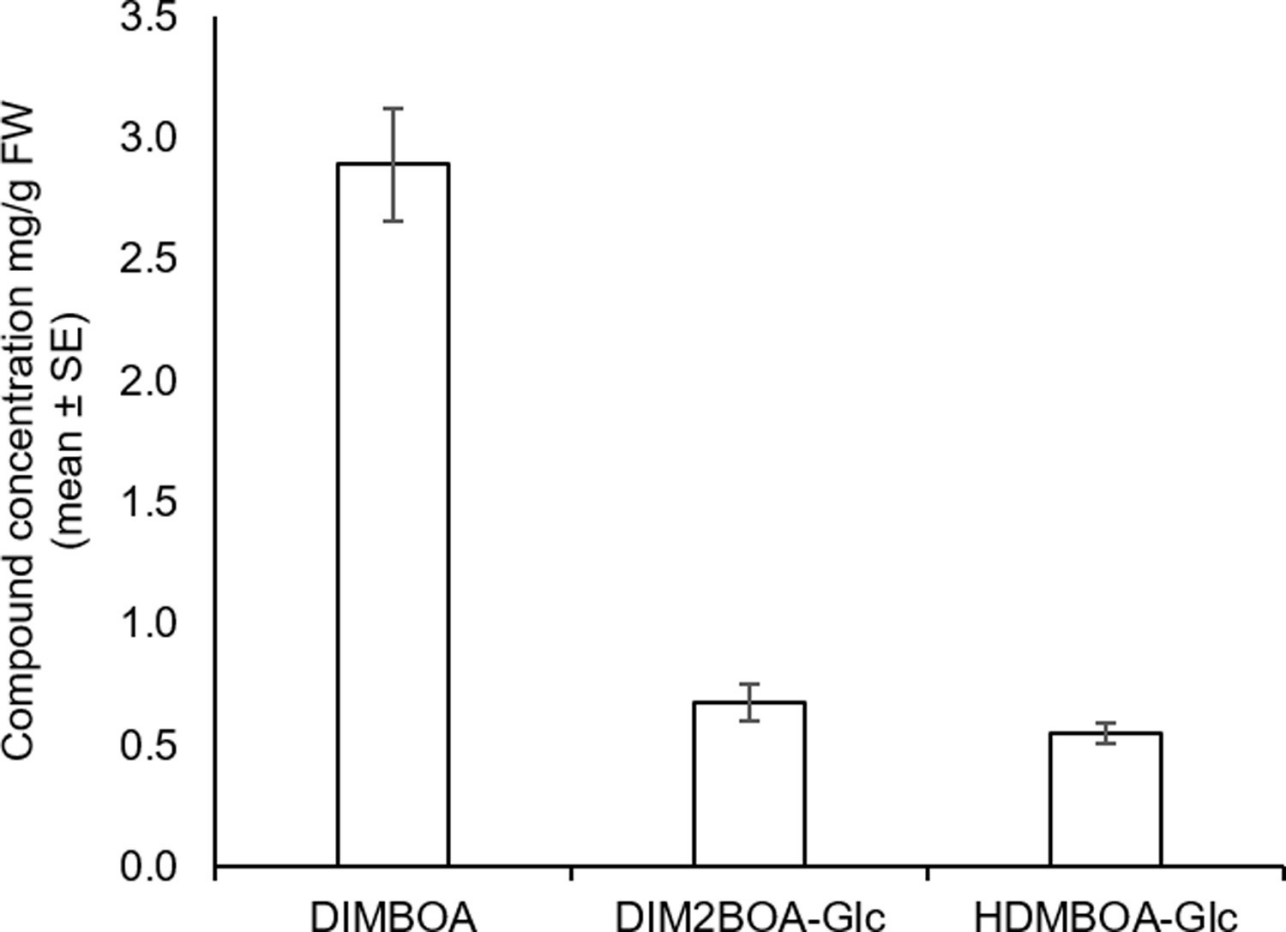
Benzoxazinoid contents in Svevo leaves. DIMBOA, DIM2BOA-Glc and HDMBOA-Glc abundance detected by HUPLC (mean ± SE; n = 5–7).

Benzoxazinoids constitute a class of plant defense compounds produced in cereals such as wheat, maize, and rye [58–60]. The benzoxazinoid metabolic pathway in maize is well characterized, while in wheat, the genes that lead from TRIBOA-Glc to DIMBOA-Glc and HDMBOA-Glc are partially known (Fig 3A). To better understand wheat metabolic responses to the different aphids’ attacks, we identified the following benzoxazinoids, DIMBOA, DIMBOA-Glc, HDMBOA-Glc and DIM2BOA-Glc, using the LC/QE/MS features, and we compared them to the standards. We calculated the fold change of these compounds relative to the untreated control (Fig 3B). We also identified ten putative aglycones of the benzoxazinoids using UV spectra and ion mass (m/z) according to the literature [47] as presented in S5 Table. As presented in Fig 3B, when analyzing the changes in the benzoxazinoid levels under aphid feeding, significant changes in DIMBOA and DIMBOA-Glc levels were detected under both *R*. *padi* and *S*. *avenae* attacks. The HDMBOA-Glc molecule was only significantly increased in response to the *S*. *avenae* and *S*. *graminum* attacks, while there was no change in the amount of DIM2BOA-Glc due to any of the aphids’ attacks (Fig 3B). We also measured the absolute amount of three benzoxazinoids from untreated Svevo leaves. This analysis revealed that the major compound is DIMBOA, which accumulates to 2.9 mg/g FW, while DIM2BOA-Glc and HDMBOA-Glc levels were lower, 0.68 and 0.55 mg/g FW, respectively (Fig 4). Together, these results reveal that although DIMBOA is only mildly induced under *R*. *padi* and *S*. *avenae* feeding (fold change of 1.34 and 1.44, respectively), it may play a major role in determining the aphids’ reproduction due to its high constitutive levels (2.9 mg/g FW). The other compounds did not show any clear pattern related to the aphid reproduction.

## Discussion

The research reveals variability in the resistance levels and the corresponding metabolic responses of the Svevo wheat to the feeding of three different aphids. The results indicated that the aphid interaction with durum wheat triggers the defense responses in different manners, ultimately affecting the aphid reproduction (Figs 1 and 2). When analyzing the progenies of three different cereal aphid pests of Svevo wheat, it is apparent that although all of them are common pests to wheat, their survival rates are different (Fig 1). The results indicated that the durum wheat accession Svevo plants are more susceptible to *S*. *graminum*, mildly resistant to *R*. *padi* and resistant to *S*. *avenae* (Fig 1). A previous study by Pereira *et al*. explored the reproductive rate of two cereal aphids reared on eight hexaploid Brazilian wheat genotypes and suggested that these wheat genotypes are more resistant to *S*. *avenae* than *R*. *padi* [17]. The variation in resistance can arise from two main adjustments: i) the aphid’s ability to attack the plant, and ii) the plant’s ability to defend itself from an aphid attack. Both plant and aphid responses are highly dependent on their genotype [17,44,61,62]. Because the plant metabolic responses were unique after applying each aphid species, we suggest that the differences in aphid reproduction occurred not only due to their intrinsic reproductive rates but also due to their interactions with the plant that triggered the plant defense responses in slightly different manners or to different extents. We further suggest exploiting the natural variation and diversity of wheat to better understand the biosynthesis of defense mechanisms and utilize them to improve resistance.

In this study, we focused on the specialized defensive compounds of benzoxazinoids. DIMBOA, the main benzoxazinoid isolated from wheat tissues, confers a toxic effect on aphids in artificial diets [41], and has also been shown to induce callose deposition as a defense response [21,32]. We demonstrated that DIMBOA and its glycosylated form, DIMBOA-Glc, slightly over-accumulated due to *R*. *padi* and *S*. *avenae* attack but were not affected by *S*. *graminum*, the aphid species to which the plants were most susceptible (Figs 1 and 3B). A previous study on the benzoxazinoid levels in several tetraploid and hexaploid wheat genotypes showed only a minor induction of DIMBOA levels and a reduction of DIMBOA-Glc levels after 24 and 48 hr of *R*. *padi* infestation [41]. We should point out that although the DIMBOA and DIMBOA-Glc levels were similarly induced under *S*. *avenae* and *R*. *padi* feeding, the Svevo wheat was most resistant to the *S*. *avenae* aphid, and it also highly accumulated HDMBOA-Glc (Figs 1 and 3B). Although DIMBOA levels were only slightly over-accumulated, it constitutes the predominant benzoxazinoid in wheat seedlings (Fig 4)[63]. The constitutive and herbivore-induced, specifically by cereal aphids, benzoxazinoid levels should be further explored to discover the corresponding genes involved in this pathway, mainly the unknown *Bx6*, *Bx7*and *Bx10-Bx14* genes.

The role of HDMBOA-Glc in plant defense against aphids is less known, although it is toxic to aphids in artificial diets [21]. It has been suggested that the main mode of action of HDMBOA-Glc is related to caterpillar resistance [64] and that caterpillar-induced methylation of DIMBOA-Glc to form HDMBOA-Glc leads to decreased aphid resistance [65]. In this work, we show that levels of HDMBOA-Glc were enhanced due to the feeding of *S*. *avenae* and *S*. *graminum* aphids, to which Svevo was the most resistant and the most susceptible, respectively (Figs 1 and 3B). Taken together, this suggests that benzoxazinoids play a role in plant resistance; however, this is not sufficient to determine resistance.

## Conclusions

In this experiment, we revealed the differential metabolic responses of durum wheat (accession named Svevo) to three different cereal aphid species. These responses include the varied resistance levels of the plant and accumulations of defense components belonging to the benzoxazinoid pathway. Our findings indicated only a mild increase in the defense metabolites DIMBOA, DIMBOA-Glc, and HDMBOA-Glc. They also indicated that DIMBOA and DIMBOA-Glc compounds are associated with aphid resistance, while HDMBOA-Glc does not have a clear function in protection against aphids. Although the data suggest that wheat defense mechanisms are complex and consist of multiple compounds acting simultaneously, it seems that benzoxazinoids may play a significant role in this interaction. We propose studying further the differences in defense responses and the roles of particular benzoxazinoids in order to understand the mechanisms by which wheat plants defend themselves from aphids and other herbivores. We also hope to examine the unknown biosynthetic enzymes of the benzoxazinoid pathway in wheat (Fig 3A marked in grey font) and to reveal additional roles played by these compounds during wheat development and interaction with the environment.

## Supporting information

S1 Table LC/QE/MS data from the negative ion mode. (XLSX)

S2 Table LC-QE-MS features from the negative ion mode that were significantly altered for at least one aphid treatment. *P* value < 0.05 FDR, fold change < 0.5 or > 2.
(XLSX)

S3 Table LC/QE/MS data from the positive ion mode. (XLSX)

S4 Table LC-QE-MS features from the positive ion mode that were significantly altered for at least one aphid treatment. *P* value < 0.05 FDR, fold change < 0.5 or > 2.
(XLSX)

S5 Table Benzoxazinoid measurements using the LC/QE/MS of the negative and positive ion modes and HUPLC. (XLSX)

## References

[1] Charmet G. Wheat domestication: lessons for the future. C R Biol. 2011 3;334(3):212–20. 10.1016/j.crvi.2010.12.013 21377616 10.1016/j.crvi.2010.12.013

[2] Peleg Z, Fahima T, Korol AB, Abbo S, Saranga Y. Genetic analysis of wheat domestication and evolution under domestication. 2011;62(14):5051–61. 10.1093/jxb/err206 PMC3193012 21778183

[3] FAOSTAT. FAO Statistical Database: Food and Agriculture Organization of the United Nations. 2014; Available from: http://faostat3.fao.org/home/E

[4] Deutsch CA, Tewksbury JJ, Tigchelaar M, Battisti DS, Merrill SC, Huey RB, et al Increase in crop losses to insect pests in a warming climate. Science (80-). 2018;919(August):916–9. 10.1126/science.aat3466 30166490

[5] Maxmen A. Crop pests: under attack. Nature. 2013 9;501:S15 10.1038/501S15a 24067760 10.1038/501S15a

[6] Rebijith KB, Asokan R, Hande HR, Joshi S, Surveswaran S, Ramamurthy VV, et al Reconstructing the macroevolutionary patterns of aphids (Hemiptera: Aphididae) using nuclear and mitochondrial DNA sequences. Biol J Linn Soc. 2017 8 1;121(4):796–814. Available from: 10.1093/biolinnean/blx020

[7] Guerrieri E, Digilio MC. Aphid-plant interactions: a review. J Plant Interact. 2008 12 1;3(4):223–32. Available from: https://www.tandfonline.com/doi/abs/10.1080/17429140802567173

[8] Rossing WAH, Van De Wiel LAJM. Simulation of damage in winter wheat caused by the grain aphid Sitobion avenae. 1. Quantification of the effects of honeydew on gas exchange of leaves and aphid populations of different size on crop growth. Netherlands J Plant Pathol. 1990;96(6):343–64. Available from: http://edepot.wur.nl/217369

[9] Bing JW, Novak MG, Obrycki JJ, Guthrie WD. Stylet penetration and feeding sites of Rhopalosiphum maidis (Homoptera: Aphididae) on two growth stages of maize. Ann Entomol Soc Am. 1991;84:549 Available from: 10.1093/aesa/84.5.549

[10] Tzin V, Fernandez-Pozo N, Richter A, Schmelz EA, Schoettner M, Schäfer M, et al Dynamic maize responses to aphid feeding are revealed by a time series of transcriptomic and metabolomic assays. Plant Physiol. 2015 11 1;169(3):1727 LP-1743. Available from: http://www.plantphysiol.org/content/169/3/1727.abstract 10.1104/pp.15.01039 26378100 10.1104/pp.15.01039 PMC4634079

[11] Zhou S, Lou Y-R, Tzin V, Jander G. Alteration of plant primary metabolism in response to insect herbivory. Plant Physiol. 2015;169(3):1488–98. Available from: 10.1104/pp.15.01405 26378101 10.1104/pp.15.01405 PMC4634104

[12] Rabbinge R, Drees EM, van der Graaf M, Verberne FCM, Wesselo A. Damage effects of cereal aphids in wheat. Netherlands J Plant Pathol. 1981 11;87(6):217–32.

[13] Nault LR. Arthropod transmission of plant viruses: a new synthesis. Ann Entomol Soc Am. 1997 9;90(5):521–41.

[14] Fereres A, Lister RM, Araya JE, Foster JE. Development and reproduction of the English grain aphid (Homoptera: Aphididae) on wheat cultivars infected with barley yellow dwarf virus. Environ Entomol. 1989 6 1;18(3):388–93. Available from: 10.1093/ee/18.3.388

[15] Blackman R, Eastop V. Aphids on the world’s crops: an identification and information guide. London: John Wiley & Sons London: John Wiley & Sons; 2000.

[16] Parry HR. Cereal aphid movement: general principles and simulation modelling. Mov Ecol. 2013;1(1):14 Available from: 10.1186/2051-3933-1-14 25709827 10.1186/2051-3933-1-14 PMC4337770

[17] Pereira JF, Sarria ALF, Powers SJ, Aradottir GI, Caulfield JC, Martin J, et al DIMBOA levels in hexaploid Brazilian wheat are not associated with antibiosis against the cereal aphids *Rhopalosiphum padi* and *Sitobion avenae*. Theor Exp Plant Physiol. 2017;29(2):61–75. Available from: http://link.springer.com/10.1007/s40626-017-0084-z

[18] Parizoto G, Rebonatto A, Schons J, Lau D. Barley yellow dwarf virus -PAV in Brazil: Seasonal fluctuation and biological characteristics. Trop Plant Pathol. 2013;38(1):11–9.

[19] Kieckhefer RW, Gellner JL. Influence of plant growth stage on cereal aphid reproduction. Crop Sci. 1988;28:688–690.

[20] Al-Mousawi AH, Richardson PE, Burton RL. Ultrastructural studies of greenbug (Hemiptera: Aphididae) feeding damage to susceptible and resistant wheat cultivars. Ann Entomol Soc Am. 1983 11 1;76(6):964–71. Available from: 10.1093/aesa/76.6.964

[21] Meihls LN, Handrick V, Glauser G, Barbier H, Kaur H, Haribal MM, et al Natural variation in maize aphid resistance is associated with 2,4-dihydroxy-7-methoxy-1,4-benzoxazin-3-one glucoside methyltransferase activity. Plant Cell. 2013;25(June):1–16. Available from: http://www.ncbi.nlm.nih.gov/pubmed/23898034 10.1105/tpc.113.112409 PMC3723630 23898034

[22] Piotr T, Narelle N, Ellen C, A. B-PN, E. CF, J. FA, et al Virus disease in wheat predicted to increase with a changing climate. Glob Chang Biol. 2015 4;21(9):3511–9. 10.1111/gcb.12941 25846559 10.1111/gcb.12941

[23] Gilabert A, Simon J-C, Mieuzet L, Halkett F, Stoeckel S, Plantegenest M, et al Climate and agricultural context shape reproductive mode variation in an aphid crop pest. Mol Ecol. 2009;18(14):3050–61. Available from: 10.1111/j.1365-294X.2009.04250.x 19538348

[24] Howe GA, Jander G. Plant immunity to insect herbivores. Annu Rev Plant Biol. 2008;59(1):41–66. Available from: http://www.annualreviews.org/doi/10.1146/annurev.arplant.59.032607.092825 18031220 10.1146/annurev.arplant.59.032607.092825

[25] Meihls LN, Kaur H, Jander G. Natural variation in maize defense against insect herbivores. Cold Spring Harb Symp Quant Biol. 2012;77:269–83. 10.1101/sqb.2012.77.014662 23223408 10.1101/sqb.2012.77.014662

[26] Bui H, Greenhalgh R, Ruckert A, Gill GS, Lee S, Ramirez RA, et al Generalist and specialist mite herbivores induce similar defense responses in maize and barley but differ in susceptibility to benzoxazinoids. Front Plant Sci. 2018;9(August):1–19. 30186298 10.3389/fpls.2018.01222 PMC6110934

[27] Niemeyer HM. Hydroxamic acids (4-hydroxy-1,4-benzoxazin-3-ones), defence chemicals in the gramineae. Phytochemistry. 1988 1;27(11):3349–58. Available from: http://linkinghub.elsevier.com/retrieve/pii/0031942288807313

[28] Feng R, Houseman JG, Downe AER, Atkinson J, Arnason JT. Effects of 2,4-dihydroxy-7-methoxy-1,4-benzoxazin-3-one (DIMBOA) and 6-methoxybenzoxazolinone (MBOA) on the detoxification processes in the larval midgut of the European corn borer. Pest Biochem Physiol. 1992;44:147.

[29] Sicker D, Frey M, Schulz M, Gierl A. Role of natural benzoxazinones in the survival strategy of plants. Int Rev Cytol. 2000;198:319–46. 10804466 10.1016/s0074-7696(00)98008-2

[30] Oikawa A, Ishihara A, Tanaka C, Mori N, Tsuda M, Iwamura H. Accumulation of HDMBOA-Glc is induced by biotic stresses prior to the release of MBOA in maize leaves. Phytochemistry. 2004;65:2995 10.1016/j.phytochem.2004.09.006 15504434 10.1016/j.phytochem.2004.09.006

[31] Grün S, Frey M, Gierl A. Evolution of the indole alkaloid biosynthesis in the genus Hordeum: distribution of gramine and DIBOA and isolation of the benzoxazinoid biosynthesis genes from *Hordeum lechleri*. Phytochemistry. 2005;66(11 SPEC. ISS.):1264–72. 10.1016/j.phytochem.2005.01.024 15907959 10.1016/j.phytochem.2005.01.024

[32] Ahmad S, Veyrat N, Gordon-Weeks R, Zhang Y, Martin J, Smart L, et al Benzoxazinoid metabolites regulate innate immunity against aphids and fungi in maize. Plant Physiol. 2011;157(1):317–27. 10.1104/pp.111.180224 21730199 10.1104/pp.111.180224 PMC3165881

[33] Mukanganyama S, Figueroa CC, Hasler JA, Niemeyer HM. Effects of DIMBOA on detoxification enzymes of the aphid Rhopalosiphum padi (Homoptera: Aphididae). J Insect Physiol. 2003;49:223 12769997 10.1016/s0022-1910(02)00269-x

[34] Castañeda LE, Figueroa CC, Fuentes-contreras E, Niemeyer HM, Nespolo RF. Energetic costs of detoxification systems in herbivores feeding on chemically defended host plants: a correlational study in the grain aphid, Sitobion avenae. 2009;1185–90. 10.1242/jeb.020990 19329751

[35] Betsiashvili M, Ahern KR, Jander G. Additive effects of two quantitative trait loci that confer Rhopalosiphum maidis (corn leaf aphid) resistance in maize inbred line Mo17. J Exp Bot. 2015;66(2):571–8. Available from: http://jxb.oxfordjournals.org/cgi/content/long/eru379v1 10.1093/jxb/eru379 25249072 10.1093/jxb/eru379 PMC4286405

[36] Frey M, Schullehner K, Dick R, Fiesselmann A, Gierl A. Benzoxazinoid biosynthesis, a model for evolution of secondary metabolic pathways in plants. Phytochemistry. 2009;70(15–16):1645–51. Available from: 10.1016/j.phytochem.2009.05.012 19577780

[37] Hanhineva K, Rogachev I, Aura AM, Aharoni A, Poutanen K, Mykkänen H. Qualitative characterization of benzoxazinoid derivatives in whole grain rye and wheat by LC-MS metabolite profiling. J Agric Food Chem. 2011;59(3):921–7. 10.1021/jf103612u 21214244 10.1021/jf103612u

[38] Adhikari KB, Tanwir F, Gregersen PL, Steffensen SK, Jensen BM, Poulsen LK, et al Benzoxazinoids: Cereal phytochemicals with putative therapeutic and health-protecting properties. Mol Nutr Food Res. 2015;59(7):1324–38. 10.1002/mnfr.201400717 25600612 10.1002/mnfr.201400717

[39] Glauser G, Marti G, Villard N, Doyen GA, Wolfender JL, Turlings TCJ, et al Induction and detoxification of maize 1,4-benzoxazin-3-ones by insect herbivores. Plant J. 2011;68(5):901–11. 10.1111/j.1365-313X.2011.04740.x 21838747 10.1111/j.1365-313X.2011.04740.x

[40] Gianoli E, Ríos JM, Niemeyer HM. Allocation of a hydroxamic acid and biomass during vegetative development in rye. Acta Agric Scand Sect B—Soil Plant Sci. 2000;50(1):35–9. Available from: 10.1080/090647100750014394

[41] Elek H, Smart L, Martin J, Ahmad S, Gordon-Weeks R, Welham S, et al The potential of hydroxamic acids in tetraploid and hexaploid wheat varieties as resistance factors against the bird-cherry oat aphid, *Rhopalosiphum padi*. Ann Appl Biol. 2013;162(1):100–9.

[42] Maag D, Köhler A, Robert CAM, Frey M, Wolfender J-L, Turlings TCJ, et al Highly localised and persistent induction of *Bx1* -dependent herbivore resistance factors in maize. Plant J. 2016;1–16. Available from: http://doi.wiley.com/10.1111/tpj.13308 10.1111/tpj.13308 27538820

[43] Sue M, Nakamura C, Nomura T. Dispersed benzoxazinone gene cluster: molecular characterization and chromosomal localization of glucosyltransferase and glucosidase genes in wheat and rye. 2011;157(November):985–97. 10.1104/pp.111.182378 PMC3252142 21875895

[44] Chandrasekhar K, Shavit R, Distelfeld A, Christensen S, Tzin V. Exploring the metabolic variation between domesticated and wild tetraploid wheat genotypes in response to corn leaf aphid infestation. Plant Signal Behav. 2018;Accepted:1–5. 10.1080/15592324.2018.1486148 PMC6110357 29944455

[45] Nomura T, Ishihara A, Yanagita RC, Endo TR, Iwamura H. Three genomes differentially contribute to the biosynthesis of benzoxazinones in hexaploid wheat. Proc Natl Acad Sci U S A. 2005;102(45):16490–5. 10.1073/pnas.0505156102 16260753 10.1073/pnas.0505156102 PMC1283429

[46] Makowska B, Bakera B, Rakoczy-Trojanowska M. The genetic background of benzoxazinoid biosynthesis in cereals. Acta Physiol Plant. 2015;37(9):176 Available from: http://link.springer.com/10.1007/s11738-015-1927-3

[47] Tanwir F, Dionisio G, Adhikari KB, Fomsgaard IS, Gregersen PL. Biosynthesis and chemical transformation of benzoxazinoids in rye during seed germination and the identification of a rye Bx6-like gene. Phytochemistry. 2017 8;140:95–107. Available from: http://www.sciencedirect.com/science/article/pii/S0031942217301693 10.1016/j.phytochem.2017.04.020 28472715 10.1016/j.phytochem.2017.04.020

[48] Robinson AG, Hsu SJ. Host plant records and biology of aphids on cereal grains and grasses in Manitoba. Can Entomol. 1963;95:134–137.

[49] Irwin ME, Thresh JM. Long-range aerial dispersal of cereal aphids as virus vectors in North America. Philos Trans R Soc London Ser B Biol Sci. 1988;321:421–46.

[50] Cilia M, Howe K, Fish T, Smith D, Mahoney J, Tamborindeguy C, et al Biomarker discovery from the top down: protein biomarkers for efficient virus transmission by insects (Homoptera: Aphididae) discovered by coupling genetics and 2-D DIGE. Proteomics. 2011;11(12):2440–58. 10.1002/pmic.201000519 21648087 10.1002/pmic.201000519

[51] Handrick V, Robert CAM, Ahern KR, Zhou S, Machado RAR, Maag D, et al Biosynthesis of 8-O-methylated benzoxazinoid defense compounds in maize. Plant Cell. 2016; 28(7):1682–1700 Available from: http://www.plantcell.org/lookup/doi/10.1105/tpc.16.00065 10.1105/tpc.16.00065 27317675 10.1105/tpc.16.00065 PMC4981128

[52] Smith CA, Want EJ, O’Maille G, Abagyan R, Siuzdak G. XCMS: processing mass spectrometry data for metabolite profiling using nonlinear peak alignment, matching, and identification. Anal Chem. 2006;78(3):779–87. Available from: https://pubs.acs.org/doi/abs/10.1021/ac051437y 10.1021/ac051437y 16448051 10.1021/ac051437y

[53] Kuhl C, Tautenhahn R, Bottcher C, Larson T, Neumann S. CAMERA: an integrated strategy for compound spectra extraction and annotation of liquid chromatography/mass spectrometry data sets. Anal Chem. 2012;84(1):283–9. Available from: https://pubs.acs.org/doi/abs/10.1021/ac202450g 10.1021/ac202450g 22111785 10.1021/ac202450g PMC3658281

[54] Tzin V, Malitsky S, Aharoni A, Galili G. Expression of a bacterial bi-functional chorismate mutase/prephenate dehydratase modulates primary and secondary metabolism associated with aromatic amino acids in Arabidopsis. Plant J. 2009;60(1):156–67. Available from: 10.1111/j.1365-313X.2009.03945.x 19508381 10.1111/j.1365-313X.2009.03945.x

[55] Xia J, Psychogios N, Young N, Wishart DS. MetaboAnalyst: a web server for metabolomic data analysis and interpretation. Nucleic Acids Res. 2009 7 1;37(Web Server issue):W652–60. Available from: http://www.ncbi.nlm.nih.gov/pmc/articles/PMC2703878/10.1093/nar/gkp356 PMC2703878 19429898

[56] Smith LACCM. Resistance to multiple cereal aphids in wheat–alien substitution and translocation lines. 2013;535–45.

[57] Voss T, Kieckhefer R, Fuller B, McLeod M, Beck D. Yield losses in maturing spring wheat caused by cereal aphids (Homoptera: Aphididae) under laboratory conditions. J Econ Entomol. 1997;90(5):1346–50.

[58] Mikić S, Shakoor A. Benzoxazinoids—protective secondary metabolites in cereals: The role and application. 2018; 55: 49–57.

[59] Niculaes C, Abramov A, Hannemann L, Frey M. Plant protection by benzoxazinoids—recent insights into biosynthesis and function. Agronomy. 2018;8(8):143.

[60] Zhou S, Richter A, Jander G. Beyond defense: multiple functions of benzoxazinoids in maize metabolism. Plant Cell Physiol. 2018 8 1;59(8):1528–37. Available from: 10.1093/pcp/pcy064 29584935

[61] Bohidar K, Wratten SD, Niemeyer HM. Effects of hydroxamic acids on the resistance of wheat to the aphid *Sitobion avenae*. Ann appl Biol. 1986;109:193–8.

[62] Loayza-Muro R, Figueroa CC, Niemeyer HM. Effect of two wheat cultivars differing in hydroxamic acid concentration on detoxification metabolism in the aphid *Sitobion avenae*. J Chem Ecol. 2000;26:2725.

[63] Ben-abu Y, Beiles A, Flom D, Nevo E. Adaptive evolution of benzoxazinoids in wild emmer wheat, Triticum dicoccoides, at “Evolution Canyon”, Mount Carmel, Israel. 2018;1–14. 10.1371/journal.pone.0190424 PMC5800564 29408917

[64] Huffaker A, Pearce G, Veyrat N, Erb M, Turlings TCJ, Sartor R, et al Plant elicitor peptides are conserved signals regulating direct and indirect antiherbivore defense. Proc Natl Acad Sci. 2013 4 2;110(14):5707 LP-5712. Available from: http://www.pnas.org/content/110/14/5707.abstract 10.1073/pnas.1214668110 23509266 10.1073/pnas.1214668110 PMC3619339

[65] Tzin V, Lindsay PL, Christensen SA, Meihls LN, Blue LB, Jander G. Genetic mapping shows intraspecific variation and transgressive segregation for caterpillar-induced aphid resistance in maize. Mol Ecol. 2015;24(22):5739–50. Available from: 10.1111/mec.13418 26462033 10.1111/mec.13418

